# Impact of gastroesophageal reflux disease on work absenteeism, presenteeism and productivity in daily life: a European observational study

**DOI:** 10.1186/1477-7525-7-90

**Published:** 2009-10-16

**Authors:** Javier P Gisbert, Alun Cooper, Dimitrios Karagiannis, Jan Hatlebakk, Lars Agréus, Helmut Jablonowski, Javier Nuevo

**Affiliations:** 1 Department of Gastroenterology, Hospital Universitario de la Princesa, Madrid, Spain; 2 Centro de Investigación Biomédica en Red de Enfermedades Hepáticas y Digestivas (CIBEREHD), Barcelona, Spain; 3 Bridge Medical Centre, Crawley, West Sussex, UK; 4 Department of Gastroenterology, Athens Medical Center, Athens, Greece; 5 Institute of Medicine, Haukeland University Hospital, Bergen, Norway; 6 Center for Family and Community Medicine, Karolinska Institutet, Huddinge/Stockholm, Sweden; 7 Klinikum Salzgitter GmbH, Salzgitter, Germany; 8 AstraZeneca, Madrid, Spain

## Abstract

**Background:**

The RANGE (*R*etrospective *AN*alysis of *G*astro*E*sophageal reflux disease [GERD]) study assessed differences among patients consulting a primary care physician due to GERD-related reasons in terms of: symptoms, diagnosis and management, response to treatment, and effects on productivity, costs and health-related quality of life. This subanalysis of RANGE determined the impact of GERD on productivity in work and daily life.

**Methods:**

RANGE was conducted at 134 primary care sites across six European countries (Germany, Greece, Norway, Spain, Sweden and the UK). All subjects (aged ≥18 years) who consulted with their primary care physician over a 4-month identification period were screened retrospectively, and those consulting at least once for GERD-related reasons were identified (index visit). From this population, a random sample was selected to enter the study and attended a follow-up appointment, during which the impact of GERD on productivity while working (absenteeism and presenteeism) and in daily life was evaluated using the self-reported Work Productivity and Activity Impairment Questionnaire for patients with GERD (WPAI-GERD).

**Results:**

Overall, 373,610 subjects consulted with their primary care physician over the 4-month identification period, 12,815 for GERD-related reasons (3.4%); 2678 randomly selected patients attended the follow-up appointment. Average absenteeism due to GERD was highest in Germany (3.2 hours/week) and lowest in the UK (0.4 hours/week), with an average of up to 6.7 additional hours/week lost due to presenteeism in Norway. The average monetary impact of GERD-related work absenteeism and presenteeism were substantial in all countries (from €55/week per employed patient in the UK to €273/patient in Sweden). Reductions in productivity in daily life of up to 26% were observed across the European countries.

**Conclusion:**

GERD places a significant burden on primary care patients, in terms of work absenteeism and presenteeism and in daily life. The resulting costs to the local economy may be substantial. Improved management of GERD could be expected to lessen the impact of GERD on productivity and reduce costs.

## Background

Gastroesophageal reflux disease (GERD) is a condition in which reflux of gastric contents into the esophagus causes troublesome symptoms such as heartburn and regurgitation and/or other complications, including reflux esophagitis [1]. In addition to esophageal manifestations, patients may also experience extraesophageal symptoms such as cough and hoarseness [2]. Current estimates suggest that GERD affects around 10-20% of the European population [3,4], with many individuals reporting marked impairment of their health-related quality of life (HRQOL) and general well-being [5-8]. Productivity, both during leisure time and while working (presenteeism), is also affected [8-12]. Associated costs can be substantial, with one US study indicating that indirect costs (as a result of presenteeism and/or due to absenteeism) accounted for 19% of the mean incremental cost of GERD to employers [13]. To date, however, few studies have evaluated the impact of GERD on productivity, and associated costs, from a European observational perspective.

The RANGE (*R*etrospective *AN*alysis of *GE*RD) study was designed to assess differences among patients consulting with a primary care physician for GERD-related reasons in several European countries. Symptom profile, diagnosis and management, as well as effects on productivity, costs and HRQOL, were examined. Here, we outline the impact of GERD on productivity as part of the RANGE study, while other results of the RANGE study are published elsewhere [14,15].

## Methods

### Study design and patients

RANGE (AstraZeneca study code: D9612L00114) was a multinational, observational programme that was conducted as a series of parallel, locally managed studies at 134 primary care sites across six European countries (Germany, Greece, Norway, Spain, Sweden and the UK). The programme was conducted in accordance with the ethical principles described in the Declaration of Helsinki, and was approved by local ethics committees.

At the start of the study, all adult subjects (≥18 years) who consulted with their primary care physician over a 4-month identification period were screened retrospectively for possible inclusion in the study (index visit). Based on medical record review, patients who had consulted at least once for GERD (with or without treatment, and regardless of whether GERD was the main reason for the visit) were identified. Patients were considered to have consulted for GERD-related reasons if they met at least one of the following criteria: they reported troublesome heartburn and/or regurgitation; GERD had been diagnosed by endoscopy (presence of esophagitis), esophageal pH monitoring (pathological esophageal pH) or by the presence of symptoms only (heartburn and/or regurgitation); GERD complications were recorded (including haemorrhage, stricture or Barrett's metaplasia); or they were prescribed proton pump inhibitors (PPIs), H_2 _receptor antagonists and/or antacids for GERD. Exclusion criteria included: prophylactic treatment with PPIs to prevent ulcers in patients taking non-steroidal anti-inflammatory drugs (NSAIDs); PPI use to heal an NSAID-induced ulcer; PPI treatment for *Helicobacter pylori *eradication; and participation in another clinical study.

From the GERD study population, a randomly selected sample was invited by letter or telephone call to participate in the study (selection of participants was made using the random number generating function of Microsoft Excel, adapted to random without replacement). Patients who agreed to participate were asked to attend a clinic visit (visit 1) at which a range of data were collected during an interview with the physician and from medical record review, including: demographics, medical history, reason for initial consultation (e.g., new symptoms in patients who had never experienced GERD symptoms previously, recurrent or persistent symptoms, follow-up visit in an asymptomatic patient) and GERD symptoms during the previous 7 days (frequency and intensity). Patients were also asked to complete the Work Productivity and Activity Impairment Questionnaire for patients with GERD (WPAI-GERD) [9,11]. This validated questionnaire uses single items to assess absence from work, presenteeism and productivity during daily life (unpaid, nonprofessional activities) in relation to reflux symptoms, with a 7-day recall period. Responses to productivity questions are graded on a 10-point scale, where higher numbers represent a greater degree of impairment.

### Statistical methods

Due to the descriptive objectives of the RANGE study, there were no hypotheses to test with statistical methods to predetermine a needed sample size. Therefore, the choice of target sample was pragmatic, based partly on the need to provide local studies with adequate power to explore the local situation and allow participating countries to fulfil their recruitment agreement. Predefined sample size for Germany, Greece, Norway and Spain was 500 patients (allowing to obtain two-sided 95% confidence intervals for single proportions using the large sample normal approximation that will extend 4.4% from the observed proportion for an expected proportion of 50% [the worst possible case]). In the same way, predefined sample size for Sweden and UK was 300 patients (allowing to obtain confidence intervals that will extend 5.7% in the worst possible case).

Reduced work productivity was measured using the WPAI-GERD questionnaire in two components: the number of hours absent from work (absenteeism) was included as one outcome measure, while the number of work hours lost due to reduced productivity while working (presenteeism) was calculated as the number of hours worked multiplied by the percentage reduction in productivity. The work time missed due to GERD (%) was calculated as [hours absent from work/(hours absent from work + hours actually worked)] multiplied by 100. The lost work productivity score was calculated as [(hours absent from work + percent reduced productivity while working multiplied by hours actually worked)/(hours absent from work + hours actually worked)] multiplied by 100.

Reflux-related productivity losses were transformed into monetary values by multiplying the number of hours lost by the most recent hourly labour cost, by country (according to Eurostat [Statistical Office of the European Communities, Luxembourg]). The monetary value of hours absent was thus calculated as hours absent from work multiplied by the hourly labour cost, and the monetary value of work hours lost due to presenteeism was calculated as work hours lost due to presenteeism multiplied by the hourly labour cost. Values are shown in Euros (€) for ease of comparison.

## Results

### Patients

Overall, 373,610 subjects consulted with their primary care physician at 134 centres over the identification period, of whom 12,815 (3.4%) did so at least once for GERD-related reasons. From the latter population a subset of 4845 randomly selected patients were invited to participate in the study; 2678 (55%) attended for consultation. The remainder were either non-contactable (n = 612), non-attendees (n = 196) or declined participation (n = 340), while 1019 patients were not invited on the basis that by-country samples sizes were reached. Demographics and clinical characteristics of participating patients, by country of residence, are presented in Table 1. The profile of patients was generally similar across the six countries surveyed; 53-61% were women and mean age was 53-60 years. Recurrence of GERD symptoms after a period of remission was the most common reason for the index visit in all countries, with the exception of Spain and Sweden (follow-up of an asymptomatic patient). Newly presenting patients with first occurrence of GERD symptoms accounted for 16.7% of the overall study population, ranging from 8% of consulting patients in Norway to 36% of consultations in Greece. Some 43% of patients were employed (including self-employed), ranging from 34% in Spain to 52% in Sweden. Demographic and clinical characteristics of such patients, by country, were generally comparable to the total patient population, with the exception that employed patients were typically younger and less likely to be women (data not shown).

**Table 1:** Characteristics of participating patients with gastroesophageal reflux disease (GERD), by country of residence

	**Germany**	**Greece**	**Norway**	**Spain**	**Sweden**	**UK**
**Number of patients**	495	505	525	477	368	308
**Women, n (%)**	295 (59.6)	265 (52.5)	303 (57.7)	280 (58.7)	223 (60.6)	171 (55.5)
**Mean age, years (SD)**	58.6 (14.5)	52.5 (14.3)	57.2 (15.2)	59.8 (15.7)	56.2 (15.0)	56.4 (15.5)
**Mean body mass index, kg/m^2 ^(SD)**	27.7 (5.1)	27.4 (4.5)	26.9 (4.7)	28.0 (4.3)	27.5 (4.8)	28.0 (5.8)
**Mean time since GERD diagnosis, years (SD)**	3.5 (3.8)	2.3 (3.0)	6.1 (7.2)	4.5 (4.7)	6.0 (8.3)	5.7 (5.8)
Employed, n (%)*	192 (38.8)	258 (51.1)	233 (44.4)	161 (33.8)	196 (53.3)	132 (42.9)
**Reason for index visit, n (%)**						
First occurrence of reflux symptoms	58 (11.7)	180 (35.6)	43 (8.2)	55 (11.7)	47 (12.8)	59 (19.2)
Recurrent symptoms after a remission period	256 (51.7)	211 (41.8)	176 (33.5)	137 (29.1)	82 (22.3)	94 (30.5)
Persistence of reflux symptoms	45 (9.1)	55 (10.9)	81 (15.4)	63 (13.4)	37 (10.1)	66 (21.4)
Follow-up visit (asymptomatic patient)	112 (22.6)	28 (5.5)	174 (33.1)	201 (42.7)	108 (29.4)	85 (27.6)
Other	30 (6.1)	15 (3.0)	51 (9.7)	15 (3.2)	94 (25.5)	8 (2.6)
**Symptoms ≥2 days/week, n (%)**						
Heartburn	81 (16.4)	213 (42.2)	86 (16.4)	158 (33.1)	50 (13.6)	NR
Regurgitation	75 (15.2)	216 (42.8)	85 (16.2)	147 (30.8)	42 (11.4)	NR
**No symptoms during previous week, n (%)**						
Heartburn	307 (62.0)	77 (15.2)	319 (60.8)	184 (38.6)	235 (63.9)	NR
Regurgitation	346 (69.9)	91 (18.0)	343 (65.9)	202 (42.3)	245 (66.6)	NR
**Moderate-to-severe symptoms, n (%)**						
Heartburn	161 (32.5)	347 (68.7)	154 (29.3)	223 (46.8)	95 (25.8)	NR
Regurgitation	124 (25.1)	289 (57.2)	127 (24.2)	204 (42.8)	91 (24.7)	NR

NR = not recorded; SD = standard deviation.

* The employed category included 9 patients for whom employment details were missing, but who were assumed to be in employment as they completed the Work Productivity and Activity Impairment Questionnaire for patients with GERD.

### Impact of GERD on productivity

#### Work productivity

**Figure 1:**
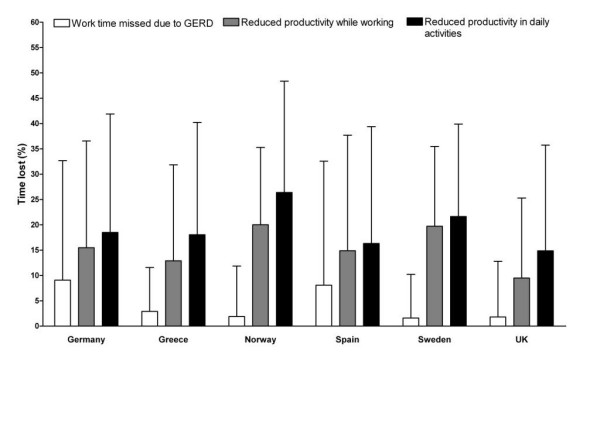
Mean (standard deviation) percent productivity lost due to gastroesophageal reflux disease (GERD), as measured using the Work Productivity and Impairment questionnaire for patients with GERD (WPAI-GERD), by country of residence

Among employed patients, the average number of work hours lost due to GERD-related absenteeism was highest in Germany (3.2 hours/week) and lowest in the UK (0.4 hours/week), with an average of up to 6.7 additional hours lost per week in Norway because of presenteeism (Table 2). The proportion of time per week lost due to GERD-related absenteeism ranged from a mean of 1.6% in Sweden to 9.1% in Germany (Figure 1). Presenteeism due to GERD led to mean losses of a further 9.5% in the UK to 20% in Norway. Data were subject to large variability, indicating that some respondents had more absences and/or severe impairment of productivity due to GERD than others.

**Table 2:** Mean (standard deviation) lost productivity due to gastroesophageal reflux disease (GERD), as measured using the Work Productivity and Impairment Questionnaire for patients with GERD (WPAI-GERD), by country of residence

		**Germany**	**Greece**	**Norway**	**Spain**	**Sweden**	**UK**
**Absenteeism, hours/week**	n	187	249	233	131	196	132
		3.2 (8.8)	1.5 (5.1)	0.9 (4.7)	2.1 (7.1)	0.7 (3.4)	0.4 (2.7)
**Presenteeism (work hours lost/week due to reduced productivity while working)**	n	158	252	231	122	178	118
		5.7 (8.6)	5.1 (7.7)	6.7 (6.2)	4.7 (8.2)	7.7 (7.5)	3.5 (6.6)
Lost work productivity score*	n	189	248	231	120	196	131
		16.7 (22.6)	12.3 (17.3)	18.1 (16.9)	17.1 (26.9)	17.0 (16.1)	9.1 (16.4)

*[(hours absent from work + percent reduced productivity while working multiplied by hours actually worked)/(hours absent from work + hours actually worked)] multiplied by 100.

Sample sizes differ for each measure due to missing data, as a small number of patients completed the WPAI-GERD questionnaire (and were therefore assumed to be employed) but did not record their professional details.

**Figure 2:**
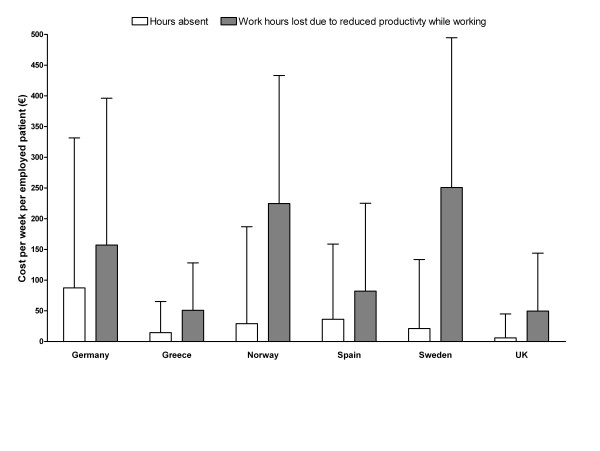
Mean (standard deviation) monetary losses related to work absenteeism and reduced productivity while working (presenteeism) due to gastroesophageal reflux disease (GERD) (transformed from Work Productivity and Impairment questionnaire for patients with GERD [WPAI-GERD]), by country of residence

The monetary impact of GERD-related work absenteeism and presenteeism was substantial in all countries (Table 3 and Figure 2). Absenteeism-related costs were greatest in Germany (mean €88/week per employed patient) and lowest in the UK (€6/week per employed patient). Presenteeism led to somewhat higher costs than absenteeism, ranging from an average of €50/week per employed patient in the UK to €251 in Sweden. Total monetary costs of hours absent plus hours lost due to presenteeism were substantial; in Sweden, for example, the mean total monetary value was €273/week per employed patient (Table 3).

**Table 3:** Mean (standard deviation) monetary losses related to work absenteeism and reduced productivity (transformed from Work Productivity and Impairment Questionnaire for patients with gastroesophageal reflux disease [WPAI-GERD] data), by country of residence

		**Germany**	**Greece**	**Norway**	**Spain**	**Sweden**	**UK**
**Sum of monetary value of hours absent and work hours lost due to reduced productivity while working, per patient per week (€)**	n	158	248	213	120	178	118
		217 (297)	65.5 (102)	251 (254)	119 (192)	273 (285)	55 (111)

#### Daily life

Reduced productivity while carrying out activities of daily life was also considerable, with patients experiencing mean productivity reductions ranging from 15% in the UK to 26% in Norway (Figure 1). Again, the data were subject to marked variability, indicating that daily life was impaired by GERD to a greater extent in some patients than in others.

## Discussion

The results of this analysis of the RANGE study show that GERD has a significant impact on patients' work productivity, in terms of absenteeism and presenteeism (decreased productivity while working). Furthermore, patients also experienced a substantial reduction in productivity in daily life. These findings, combined with the considerable impact on patients' HRQOL observed in the RANGE programme [14], help us to further understand the burden associated with this disease. A structured approach to management of GERD, tailoring therapy according to patient need, may lessen this impact on productivity and, in turn, reduce costs. One way to achieve this might be to employ management tools such as GerdQ [16], which evaluates the frequency of GERD symptoms, sleep disturbance and use of over-the-counter medication for heartburn and/or regurgitation. In turn, physicians would be better able to quantify the impact of GERD and tailor treatment accordingly.

Generally, Norway, in particular, as well as Sweden, had the highest figures for reduced productivity while working (presenteeism) and in daily life, and work time missed due to GERD symptoms. The lowest figures for all productivity variables were consistently observed in the UK. However, it should be noted that the data observed in this report are subject to considerable variation between the European countries surveyed. Overall, productivity losses reported in this study are higher than those reported in some previous studies. For example, data from a 2004 survey of US respondents with self-reported symptoms of GERD, of whom 58% were employed, reported a 7.5% reduction in work productivity and 0.9 hours of absenteeism per week [8], compared with up to 20% and 3.2 hours, respectively, in our study. This difference most likely reflects the use of the generic, rather than GERD-specific, version of the WPAI in the 2004 US survey and internet-based recruitment of respondents with self-reported GERD symptoms [8], rather than physician-diagnosed patients with GERD. Indeed, our results are similar to previous studies in which productivity losses were measured using the WPAI-GERD questionnaire [9,11]. In one study, GERD symptoms led to 2.5 hours absent from work, 23% reduced productivity due to presenteeism and 30% reduced productivity in activities of daily life [9].

It is of interest to compare GERD-related productivity impairments in the RANGE study with findings from other studies in patients with chronic disorders, which have used modified versions of the WPAI questionnaire. For example, in a study that used the allergy-specific version of the WPAI questionnaire among a sample of patients with allergic rhinitis, up to 40% of work time was lost due to presenteeism (compared with 10-20% in RANGE) and a loss of up to 50% in productivity during daily activities was apparent (compared with 15-26% in RANGE) [17]. However, no loss of work time due to absenteeism was reported in this study (compared with 2-9% in RANGE). Another study that used a version of the WPAI modified for irritable bowel syndrome reported productivity reductions of 6% due to absenteeism, 31% due to presenteeism and a 37% impairment in daily activities [18]. Further, a study that used the Crohn's disease version of the WPAI found that patients with this disease missed 18% of work time due to absenteeism and > 40% due to presenteeism, in addition to a 52% impairment in daily activities [19].

Several limitations need to be considered with regard to the RANGE study. For example, patients were randomly selected from those consulting for a number of GERD-related reasons, including asymptomatic patients undergoing routine follow-up. While the RANGE study population therefore reflected the heterogeneous nature of GERD in primary care, the inclusion of asymptomatic patients may have served to underestimate the true impact of GERD on productivity and associated costs (this may explain why productivity impairment was not as marked as for other chronic diseases, as discussed above). Further investigation of the differences in productivity impact and costs between asymptomatic patients, and those consulting because of symptomatic GERD (including recurrent, persistent or newly occurring symptoms), may be warranted. The heterogeneous nature of the population included in the RANGE study means that many individuals would have been included who do not seek treatment for their GERD symptoms and may therefore not be correctly diagnosed; the productivity impairment in such individuals may also be noteworthy. In addition, the WPAI-GERD questionnaire, while being validated in English and Swedish, has not been validated in German, Greek, Norwegian or Spanish, placing a potential limitation on the accuracy of the data gathered from respondents from these countries. One should also consider the limitations of retrospective and observational studies such as RANGE, in terms of recall bias and difficulties with estimating productivity losses based on subjective reports. It is possible that the 7-day recall period used in the WPAI-GERD may also lead the patients, in whom significant episodes of GERD may only occur every few weeks, to underestimate the impact of this disease on productivity. The impact of GERD may also vary according to the type of work and differences in hourly labour costs, which may account for observed between-country differences in costs related to decreased productivity. Comparisons between countries for monetary transformations should be made with this in mind. Another limitation of the latter analysis is that data are presented in terms of weekly costs; this assumes that the productivity impact of GERD is stable over time, which is likely to not be the case given the typical course of the disease. The inherent limitations associated with calculating monetary losses based on reported labour costs should also be considered along with societal differences between the European countries surveyed in RANGE. Different social security systems, for example, may partly account for the wide variability of data observed in this study.

## Conclusion

GERD accounts for a significant burden on primary care patients, in terms of work absenteeism and decreased productivity both while working (presenteeism) and in daily life. The costs to the local economies as a result of GERD-related absence from work and reduced productivity while working may be substantial across the European countries. Improved management of GERD, with tailoring of therapy to specific patient needs, could be expected to lessen the impact of GERD on productivity, thereby reducing costs.

## Abbreviations

GERD: gastroesophageal reflux disease; HRQOL: health-related quality of life; NSAID: non-steroidal anti-inflammatory drug; PPI: proton pump inhibitor; WPAI-GERD: Work Productivity and Activity Impairment Questionnaire for patients with GERD.

## Competing interests

JPG has received educational/research grants and consulting fees from AstraZeneca; AC has no competing interests to declare; DK has received research grants from Abbott and speaker fees from Janssen, AstraZeneca and Falk (Galenica); JH has received speaker fees from AstraZeneca; LA has received research grants and speaker fees from AstraZeneca, and is a former advisory board member for Orexo AB; HJ has received speaker fees from AstraZeneca; JN is an employee of AstraZeneca.

## Authors' contributions

All authors were involved in data interpretation and manuscript preparation. Data analysis was provided by AstraZeneca. All authors read and approved the final submission.
